# Toxicology

**Published:** 2008-10

**434**

**Hepatoprotective effects of *Polygonum bistorta* and active principle on albino rats intoxicated with carbon tetrachloride**

Mittal Deepak Kumar, Jaswal Amita, Shukla Sangeeta

**Reproductive Biology and Toxicology Laboratory, School of Studies in Zoology, Jiwaji University, Gwalior (M.P.), India.**

Herbal preparations have been recommended in alternative systems of medicine for the treatment of hepatic disorders. Among the various studies involved in hepatotoxic effect of CCl_4_ (1.5 ml/kg, *i.p*.) and paracetamol (2 g/kg, *p.o*.) is oxidative damage through free radical generation. Drug-induced hepatotoxicity is still a significant unresolved clinical problem as liver is the most common site of damage. *Polygonum bistorta* is powerful astringent, demulcent, diuretic, febrifuge, laxative, strongly styptic and rich in tannins. No systematic study has been done on protective efficacy of *Polygonum bistorta* (PB) and its active principle Tannic acid (TA) to treat hepatic diseases. The goal of the present work is to evaluate and compare the efficacy of root extract of *Polygonum bistorta* (100 mg/kg) and tannic acid (25 mg/kg, *p.o*.) against toxicants induced damage in liver and kidney. The activities of transaminases, alkaline phosphatase and protein were increased in serum after 48 h days of toxicants administration A significant rise was observed in lipid peroxidation level however reduced glutathione content was decreased. A concomitant fall was observed in the enzymatic activities of adenosine triphosphatase, glucose-6-phosphatase. Administration of PB and Ta significantly brought the values of studied parameters towards normal and also reversed the histopathological alterations in liver and kidney. Thus, it may be concluded that *P. bistorta and* tannic acid can be used to reduce the hepatorenal damage and may serve as an alternative medicine.

**435**

**Cotherapy of chelating agents and Ca/Fe against aluminium induced nephrotoxicity**

Shrivastava Sadhana, Shukla Sangeeta, Mathur Ramesh

**Reproductive Biology and Toxicology Laboratory, School of Studies in Zoology, Jiwaji University, Gwalior-474011, India.**

The toxicity of Aluminum (Al) can be traced to increased deposition in bone and the central nervous system, particularly in the presence of reduced renal function. This study aimed at evaluating the protective effects of combination therapy on histopathological and biochemical parameters in kidney of rats. Al(NO_3_)_3_ (5mg/Kg/day, *i.p*.) was administered to rats for 90 days, followed by therapy with NAC/ HEDTA+Ca+Fe for 7 days. Al generates oxidative stress in kidney which caused significantly rise were observed in the activities of creatinine, urea, serum alkaline phosphatase, uric acid and BUN whereas serum protein was found to be decline after toxicant exposure. TBARS levels, acid phosphatase were significantly higher and total glutathione content were significantly lower in renal toxicity. Decrease in activities of succinic dehydrogenase, adenosine triphosphatase, glutathione reductase and glutathione-6-phosphatase dehydrogenase in kidney were noted. The tissue retention of Al varied among the organs. Animals showed significantly higher levels of Al in kidney when compared to control animals. Co therapy was significantly effective in removing Al from the organs and showed over all improvement in all the biochemical changes. TEM of kidney after Al exposure represents degeneration in mesangial cell, distorted filtration membrane, reduced no of foot processes, disruption of endoplasmic reticulum, vacuolization and degenerated mitochondria of necrotic cells. Proximal tubules revealed damaged microvilli. Conjoint treatment showed rounded nuclei, elongated mitochondria with clear seen crests. Epithelial cell of tubule exhibits abundant microvilli. Treatment with HEDTA+Ca+Fe was more effective when compared to NAC+Ca+Fe.

**436**

**Resveratrol ameliorates carbon tetrachloride induced acute liver injury in mice**

Bhadauria Monika, Nirala Satendra Kumar, Shukla Sangeeta

**Reproductive Biology Laboratory, School of Studies in Zoology, Jiwaji University, Gwalior- 474011, India.**

In the present investigation, an attempt has been made to validate the hepatoprotective potential of resveratrol (30 mg/kg, p.o.) in mice following different routes (p.o. and s.c.) of exposure to carbon tetrachloride (CCl4, 1.0 ml/kg). Administration of CCl4 caused significant increase in the release of transaminases, alkaline phosphatase, lactate dehydrogenase, γ-glutamyl transpeptidase, creatinine kinase, total bilirubin, urea and uric acid in serum. Significantly enhanced hepatic lipid peroxidation and oxidized glutathione with marked depletion of reduced glutathione were observed after 24 h of CCl4 intoxication. It was also found that CCl4 administration caused severe alterations in hepatic histoarchitecture. Hepatic injury was more severe in those animals who received CCl4 by oral route than those who exposed to CCl4 subcutaneously. Resveratrol treatment was capable of reversing hepatic damage induced by acute intoxication of CCl4 and showed pronounced curative effect against lipid peroxidation and deviated serum enzymatic variables as well as maintained glutathione status toward control. Treatment with resveratrol also recovered the CCl4 induced histopathological alterations in liver. The results of the present study suggest that resveratrol has potential to exert curative effects against experimentally induced liver injury.

**437**

**Cut across the wall: Cell to cell communication through intercellular bridges**

Nirala Satendra Kumar, Bhadauria Monika, Mathur Ramesh

**School of Studies in Zoology, Jiwaji University, Gwalior-474011, India.**

Cell to cell communication is essential to coordinate developmental events and functions of multicellular organisms. Chemical signals are transmitted between cells via intricate structures such as gap junctions and chemical synapses. Another mechanism of intercellular communication has recently been proposed in various organisms that involves “Intercellular Bridges” and “Tunneling Nanotubules” *ex vivo*. Much of the information on the mode of cellular exchange across these tubular structures comes particularly from germ cells, in which intercellular bridges are supposed to be responsible for their synchronous development. Intercellular bridges can serve as a conduit for exchange of cellular material. In contrast to gap junctions that permit direct passage of small molecules up to 1 kDa between cells, intercellular bridges provide for exchange of larger materials including organells, nutrients and other cytoplasmic constituents. Molecules involved in death inducing mechanism may spread through intercellular bridges leading to apoptosis of chains of interconnected cells. Quite recently, specific association of such bridges with malignant transformation *in vitro* and their potential for supporting cancerous proliferation *in vivo* has also been proposed. During carcinogenesis, there are significant changes in intercellular communication, altering the balance between cell proliferation and death. To date, much of the research on this topic has focused on gap junctions. Restoration of appropriate intercellular communication distorted by carcinogenesis may be a novel approach to cancer treatment. In cancer cells with deficient gap junctions, intercellular bridges may have a prominent role in intercellular communication and, as such, may be useful targets for anti-cancer therapies.

**438**

**Hepatoprotective efficacy of Majoon Dabeed-ul-Ward against experimentally induced liver injury in mice**

Shakya K Arvind, Sharma Samta, Shukla Sangeeta

**Reproductive Biology and Toxicology Lab, School of Studies in Zoology, Jiwaji University, Gwalior 474011, MP, India.**

Majoon Dabeed-ul-Ward (MD) is a polyherbal formulation consisting of 19 medicinal plants which are derived from the traditional Unani system of medicine in India. This study was conducted to investigate the protective effects of polyherbal formulation of Majoon Dabeedul-Ward (MD) (1033 mg/kg, *p.o*, once only for 5 days) against alcohol (10%, 20%, 30%, 40% in I, II, III, IV and V week with drinking water) induced oxidative damage in rats. Alcohol induced liver damage significantly (*P* < 0.05) increased the activities of transaminases (AST / ALT), and alkaline phosphatase (ALP) and level of albumin, urea, uric acid and creatinine in serum and decreased the activities of reduced glutathione (GSH) content in liver whereas increased hepatic malondialdehyde (MDA) content as compared with control group. Treatment with MD could significantly (*P* < 0.05) decrease the ALT, AST, and ALP, albumin, urea, uric acid, triglyceride, cholestrol, HDL and LDL and creatinine levels in serum and increase the activities of GSH content and decrease the MDA content in liver when compared with alcohol treated group. The results suggest that MD exhibits potent hepatoprotective effects on alcohol-induced liver damages in mice, and that the hepatoprotective effects of MD may be due to both the increase of antioxidant enzymes activities and inhibition of lipid peroxidation.

**439**

**Ameliorative effect of butea monosperma on model hepatotoxicant-induced damage**

Sharma Neetu, Raghuvanshi Suchita, Shukla Sangeeta

**Reproductive Biology and Toxicology Lab, School of Studies in Zoology, Jiwaji University, Gwalior 474011, MP, India.**

***Butea monosperma*** Lam. (Fabaceae) is well documented medicinal plant, which is used in *Ayurvedic* system for liver ailments. It is traditionally employed intensively as folklore remedy for a wide spectrum of liver diseases in India. It is reported to have chemoprotective, anti inflammatory, anti diabetic, antistress and antimicrobial activities. Aqueous extract of the flower of the *Butea monosperma* was evaluated at different dose levels (208, 416 and 832 mg/kg) for its hepatoprotective efficacy to validate its use in traditional system of medicine. In the present study, hepatoprotective efficacy of *Butea monosperma* was evaluated against CCl_4_ induced acute (1.5ml/kg, *i.p*.,) and subchornic (0.15ml/ kg, *i.p*.) study. *In vivo* efficacy of acute study was reinforced hepatoprotective effect by decreasing the activity serum enzyme, protein, albumin and lipid peroxidation while it significant increased the reduced glutathione levels of tissue. The results indicated that *Butea monosperma* showed protection at the does 832 mg/kg. Subchronic exposure to CCl_4_ (21 days) showed its toxic effect on various blood and tissue biochemical indices. Therapy of *Butea monosperma* at the dose 832 mg/kg for 5 days showed its protective effect on DNA damage and histopathological observations. The results obtained indicate that *Butea monosperma* is a potential source of natural hepatoprotectives and free radical scavenger to comparable to silymarin.

**440**

**Antioxidant and hepatoprotective activity of aqueous extract of rosa damascena against CCL4-induced oxidative damage**

Saxena Monika, Sharma Yamini, Shukla Sangeeta

**Reproductive Biology and Toxicology Lab, School of Studies in Zoology, Jiwaji University, Gwalior 474011, MP, India.**

***Rosa damascene*** is widely cultivated ornamental plant. It has several therapeutic effects, including calmative, ant anxiety, antioxidant and hepatoprotective and also used in respiratory disorders, tonsillitis, headache and eye disorders. The present investigation was planned to evaluate the hepatoprotective activity of aqueous extract of *Rosa damascena* flower (513 and 1026 mg/kg, p.o., for 5 days) against CCl_4_ (0.15ml/kg, *i.p*., for 21 days) induced hepatotocellular damage in rats. The administration of toxicant altered blood biochemical variables and significantly increased the level of serum marker enzymes such as AST and ALT. Hepatic and renal lipid peroxidation was increased significantly, whereas substantial depletion was observed in enzymatic antioxidants (glutathione reductase and glutathione peroixidase) and reduced glutathione level. A slight elevation was found in protein content, while a noteworthy fall was observed in the activities of aniline hydroxylase, adenosine triphosphatase and glucose-6-phosphatase after induction of toxicity. In addition, the CCl_4_-induced pathological changes in liver were evaluated by histopathological studies. Treatment with the aqueous extract of *Rosa damascene* at a dose of (513 mg/kg) significantly altered these changes to almost normal values. Histological analysis showed that the extract significantly ameliorated the hepatic injury. Therefore, our findings provide evidences that extract could protect liver against the CCl_4_-induced oxidative damage and this hepatoprotective effect might be contributed to its modulation on detoxification enzymes and its antioxidant and free radical scavenger effects.

**441**

**Analysis of acute poisoning cases reported to national poisons information centre (NPIC) (1999-2008)**

Srivastava Amita, Peshin S Sharda, Halder Nabanita, Gupta YK

**Department of Pharmacology, All India Institute of Medical Sciences, New Delhi - 110029, India.**

Acute poisoning is a common medical emergency that carries a high mortality and morbidity rate in India. Due to easy availability and low cost, the use of chemical agents in industries, agricultural and domestic sectors has increased, which has resulted in frequent incidences of accidental/intentional poisonings. The present analysis was carried out to determine the trends of poisoning based on telephonic inquires along with various parameters which included mode of poisoning, type of poison used, route of exposure, most vulnerable age groups and outcome of poisoning. A retrospective analysis of poisoning calls received by the NPIC (April1999-March 2008) showed a total of 9453 cases from all over India of acute poisoning during a period of nine years. The call details include the time, date, mode of inquiry, enquirer’s name and address, patients or victim’s age, sex, symptoms and treatment given. Of the total calls, 92% were on poisoning management and 8% seeking information on various poisons and availability of antidotes. The age ranged from less than one year to 70 years, with the highest incidence in the range of 15-45 years and the males (58.5%) outnumbering the females (41.5%) The most common mode of poisoning was intentional (51.5%) followed by accidental (46.3%) and the other modes of poisoning contributed 2.1%.The most common route of exposure was oral (94.6%). The results revealed that the poisoning agents belonged to various groups like household products, agricultural products, drugs, industrial chemicals, environmental toxins, miscellaneous and some remained unknown. The incidence of poisoning was highest in household agents (42.5%) followed by drugs (19.2%), agricultural pesticides (13.2%), industrial chemicals (10.9%), animal bitesand stings (4.3%) plants (2.3%) unknown (3.6%) and miscellaneous groups (3.9%).The study highlighted that household products were the main agents implicated in poisoning; therefore, efforts should be taken to create awareness among people regarding the judicious use of chemicals at home and agricultural sector.

**442**

**Pesticide poisoniing trend from data reported to National Poisons Information Centre, AIIMS, New Delhi**

Halder Nabanita, Peshin Sharda S, Srivastava Amita, Gupta Yogendra Kumar

**National Poisons Information Centre, Department of Pharmacology, All India Institute of Medical Sciences, New Delhi, India.**

Pesticide poisoning is a major health concern today in developing world. Due to the extensive use and easy availability, pesticides are commonly consumed for intentional self-poisoning. The present study highlights the trend of acute poisoning by agricultural pesticides as well as by household pesticides. A total number of 2877 cases were reported to National Poisons Information Centre, Department of Pharmacology, All India Institute of Medical Sciences, New Delhi during the period of 8 years (April 1999 -March 2007) from all over India. Acute poisoning due to household pesticides comprised of 1890 cases where as the number of poisoning cases due to agricultural pesticides was found to be 987. Surprisingly, the common mode of poisoning in the both the categories were suicidal with males outnumbering females. Amongst household products, pyrethroids showed highest incidence (35.55%) followed by rodenticides (29.1%), organophosphates (6.77%) and carbamates (6.19%). On the other hand, amongst agricultural products maximum poisoning was reported due to aluminium phosphide (31.3%) followed by organochlorines (24.92%), organophosphates (22.49%), ethylene dibromide (10.13%), herbicides and weedicides (5.57%) and least with carbamates (0.506%). The accidental poisoning was mainly found to be in children (1-14 years). The results emphasize the importance of developing an urgent poisoning prevention programme with a special focus on improving clinical services, restricting the use of highly toxic pesticides as well as initiating farmers education programme focussing on safe pesticide practices at primary care level.

**443**

**Role of α- lipoic acid (ALA) in amelioration of arsenic induced behavioral, biochemical and morphological alterations in rats**

Dhar P, Dixit S, Mehra RD

**All India Institute of Medical Sciences, New Delhi-29, India.**

The ubiquitous distribution of arsenic in the environment is being perceived as a matter of global concern. The present work was planned to study the ameliorative efficacy of α -lipoic acid (ALA) on arsenic induced alterations in behavioral, biochemical and morphological parameters in rats. Mother reared Wistar rat pups were divided into the control (receiving no treatment and those receiving distilled water) and the experimental groups (n=10/ subgroup). Sodium arsenite alone (1.5 and 2.0 mg/kg body wt.) and with ALA (70 mg/kg body wt.) was administered intraperitonealy (i.p.) from PND 4 to PND 15 to experimental groups. On PND 14–16, elevated plus maze test was performed to determine the retention transfer latency. Half of the animals were sacrificed by cervical dislocation on PND 16 and the hippocampus was dissected out on ice and processed for estimation of glutathione (GSH) and superoxide dismutase (SOD) following the standard protocol. The remaining half were perfusion fixed with 4% paraformaldehyde and the brain was removed. The tissue blocks containing hippocampus were processed for paraffin embedding and 8*µ*m thick paraffin sections were stained with cresyl violet and observed under the microscope. The observations revealed a) significant decrease of retention transfer latency on PND 16 and significant increase in GSH and SOD levels b) normal cell density and organization of pyramidal cell layer in cornu ammonis region of hippocampus in animals receiving ALA with sodium arsenite. These findings are suggestive of ameliorative efficacy of ALA against arsenic induced oxidative stress in the central nervous system of rat.

**444**

**Assessment of awareness of environmental mercury pollution in healthcare system**

Gaurav Rohit, Kumar Gajendra, Chauhan Anjali, Peshin Sharda Shah, Halder Nabanita, Gupta Yogendra Kumar

**Department of Pharmacology, All India Institute of Medical Sciences, New Delhi - 110029, India.**

Mercury, a ubiquitous environmental toxin is extensively used in healthcare setup. A questionnaire based survey was done in different Departments (Medicine, Dentistry, Emergency Services, Pathology, Surgery, and Pediatrics) of the All India Institute of Medical Sciences, New Delhi, to assess the awareness amongst healthcare professionals about the proper handling and disposal of mercury containing items. All the questions were based on knowledge, use and disposal of mercury based products. The survey included 150 subjects (Doctors 50, Nurses 50, and Technicians 50). Evaluation was done on the basis of marks obtained by the subjects against 25 multiple choice questions (Score >50% was considered as good, 25-50% as satisfactory and <25% as poor performance). The results showed 12% doctors scored >50% marks. Majority of them (86%) scored satisfactory and, just 2% secured <25% marks. On the other hand, only 2% nurses obtained marks >50%, whereas satisfactory and poor performance was shown by 76% and 22% respectively. Among the technicians just 6% scored >50% marks. The performance of 36% was found to be satisfactory and majority of them (58%) scored less than 25% marks. Statistical analysis showed that the average awareness is more, though not significant in doctors in comparison to the other two counterparts. The awareness was found to be more in nurses as compared to technicians. Low level of general awareness highlighted the lacunae in knowledge regarding the mercury environmental pollution. Proper strategies must be formulated and implemented to increase the general awareness. Further, stress needs to be laid on proper handling and disposal to reduce the mercury load in environment.

**445**

**Hyperlipidemia enhanced ischemia reperfusion induced renal damage in rats**

Bhalodia VR^1^, Bhalodia YS^2^, Vaghasiya JD^2^, Jivani NP^2^, Kanzariya N^1^, Patel RK^1^, Patel NJ^1^

**^1^S.K.Patel College of Pharmaceutical Education and Research, Ganpat University, Kherva, Dist-Mahesana, Gujarat, ^2^R.B.Patel Mahila Pharmacy College, S.N.D.T Women’s University, Atkot, Rajkot, Gujarat, India.**

The effects of hyperlipidemia on ischemic reperfusion induced renal damage were evaluated in rats. In the experimental methodology wistar rats were fed a regular rat diet (ND) or a high fat diet (HC) supplemented with 3% cholesterol, 20% coconut oil, 10% glucose, 0.3% Dl Methionine and 0.2% NaCl for 4 weeks. At the end of the fourth week, the animals were anesthetized and submitted to a suprapubic incision. Ischemic renal failure was induced by clamping the both renal artery. After 60 min, the clamp was removed and underwent for 24 hrs reperfusion period. At the end of the reperfusion period, blood was drawn to study serum creatinine, urea, uric acid, blood urea nitrogen (BUN) level and lipid profile, and a bilateral nephrectomy was done to determine superoxide dismutase (SOD), catalase (CAT), reduced glutathione (GSH), lipid peroxidation (MDA) levels and histological study in renal tissue. HC rats shows significant rise in body weight and serum lipid profile like total cholesterol, triglyceride, LDL, VLDL cholesterol when compared with ND rats. Serum creatinine, urea and BUN were higher in HC-rats than in ND-rats indicating that hyperlipidemia potentiated renal ischemic injury. Antioxidant enzymes were depleted in HC rats compared to ND rats indicating generation of oxidative stress. These data suggest that oxidant stress contributed to the deleterious effects of hyperlipidemia on the renal ischemia reperfusion injury.

**446**

**Comparative toxicity study of indomethacin a non steroidal anti-inflammatory drug with indolizine nucleus for ulcerogenic effect**

Chakraborty A, Baidya M

**Krupanidhi college of Pharmacy, Bangalore, India.**

Indomethacin inhibits both COX-1 and COX-2, it inhibits the production of prostaglandins in the stomach and which maintain the mucus lining of the gastro intestinal tract. Indomethacin, therefore, like other nonselective COX inhibitors, can cause peptic ulcer. The ulcers can result in serious bleeding and/or perforation requiring hospitalization of the patient. Some even die from these complications. A single application of the indomethacin in the dose of 60 mg per kilogram of body weight had an ulcerogenic action in 100% of cases. Changes in the mucous coat of the small intestine testified to the development of inflammation. The present study includes the synthesis of an indolizine nucleus which has similar structure with indole nucleus present in indomethacin and fused with essential amino acid. The synthesize compounds were studied pharmacologically for analgesic on albino mice by Eddy’s hot plate method, and anti-inflammatory on wister rat by producing carageenin induce edema which shows the similar (1a, 1c, 1e) or better(1b, 1f) effect with compared to indomethacin. The ulcerogenic activity was done on wister rat showing significant reduction on gastric secretion compared to indomethacin. Based on above study it has been concluded that the changing the nucleus of indomethacin by indolizine may reduce the toxic effect of indomethacin.

**447**

**Strategies for safe and effective therapeutic measures against mercury induced oxidative stress**

Singh Varsha^1^, Shukla Sangeeta^2^, Gupta YK^1^

**^1^All India Institute of Medical Sciences, New Delhi - 110029, ^2^Trace element- Institute for UNESCO And Reproductive Biology and Toxicology Laboratory, School of Studies in Zoology, Jiwaji University, Gwalior, India**

Mercury exposure is the second-most common cause of metal poisoning. It is quite stable and biotransformed to a highly toxic metabolites thus eliciting morphological and biochemical alterations. The over all protection of cells from oxidative damage is mediated by enzymatic and non-enzymatic systems. The aim of the present study is to evaluate the efficacy of thiol containing chelating agent in reducing CH_3_Hg^+^ from brain and blood mercury levels following prolonged dimethyl mercury (1 mg/kg, p.o., 12 weeks) exposures in rats. The injury produced by dimethyl mercury is associated with a variety of biochemical abnormalities as evident in the present investigation. Hg induces alterations in the cell membrane permeability and inhibition of ion transporting system. Significant rise in the LPO is one of the primary effects induced by oxidative stress and occurs readily in the tissues resulting in significant amount of the transaminases, LDH, and GGT in the serum. GSH contents decreased after Hg administration which is also responsible for the decrease in the enzymes of redox cycle (GR and GPx) and AChE in fore, mid and hind brain. Animals also showed significantly higher levels of mercury in brain and blood. The metal-SH (mercury-GSH) conjugation process is desirable in that it results in the excretion of the toxic metal and the combination of the glutathione (0.30 mM/kg, i.p.) and magnesium (10 mg/kg, p.o.) showing synergistic effect to remove mercury. Thus the combination has significant antioxidant potential, which provides protection against deleterious metal mediated free radical attacks thereby preventing oxidative degradation of biological membrane.

**448**

**Is genotoxicity induced by metronidazole reversible in rodents: An observation?**

Singh S, Sharma R, Singh GD, Khajuria A, Kaul A, Singh SK, Taneja SC, Saxena AK, Johri RK

**Indian Institute of Integrative Medicine (CSIR), Canal Road Jammu Tawi (JandK) - 180001, India.**

Metronidazole (MTZ) is one of the world’s most widely used drugs for the treatment of anaerobic infections and the treatment of choice for most patients with diarrhea. It is a nitroimidazole moiety, gets converted in the anaerobic organisms by the redox enzyme pyruvate-ferredoxin oxidoreductase. The nitro group of MTZ is chemically reduced by ferredoxin and the products are responsible for disrupting the DNA helical structure thus inhibiting nucleic acid synthesis. MTZ exerts its antimicrobial effects through the production of free radicals, which are toxic to the microbes. Previous toxicological and genotoxicological studies of MTZ revealed that long term administrations of high doses of some 5- nitroimidazole derivatives to rats and mice resulted in various tumors. Genotoxicological studies of MTZ through the stages of the seminiferous epithelial cycle and spermatozoa morphology reveled that it has to be considered as a conceivable thread regarding male infertility. The aim of this study is to assess reversal of the morphological sperm abnormalities after drug treatment. Swiss mice were used to carry out *in vivo* study. Three groups of animals each of 10 male swiss mice were taken. First and second group were given two different doses of MTZ at 130 mg/kg and 200 mg/ kg i.p. for 5 days. Third group kept as normal control and given only vehicle. Animals were sacrificed after 5, 10 and 30 days of treatment to observe reversal in sperm morphological abnormalities. The results observed on the basis of data obtained revealed that 23.78 % reversion in the abnormality on the day 10th and 72.48% on day 30th for 130 mg/kg whereas for 200 mg/kg dose it was 13.83% on day 10th and 32.58% on 30th day. The conclusion of the study is still awaited because of confirmation.

**449**

***In vivo* genotoxicity evaluation of a plant based antiarthritic and anticancer therapeutic agent boswellic acids in rodents**

Singh S, Sharma R, Singh GD, Khajuria A, Kaul A, Singh SK, Taneja SC, Saxena AK, Johri RK

**Indian Institute of Integrative Medicine (CSIR), Canal Road Jammu Tawi (JandK) 180001, India.**

The genotoxic potential of anti-inflammatory / anti-arthritic and anticancer plant based drug molecule Boswellic acids (BA) was studied in the *in vivo* system. Systematic literature survey revealed that studies on the ability of boswellic acids to affect germ cell DNA are not available. The general toxicity study of BA has been already conducted by us which revealed it to be non toxic in acute and chronic toxicity studies in rodents and primates up to 2000mg/kg. The genotoxicity was carried out in Wistar rats using different cytogenetic assay system abnormalities. This study was aimed to investigate various cytogenetic abnormalities viz., chromosomal aberrations, comet assay, sperm morphology assay and micronuclei assay. Five groups of animals each comprised of five rats were taken for each study. First, second and third groups were given the test drug BA at 125, 250 and 500mg/ kg p.o., prepared as fine homogenized suspension in 2% gum acacia, fourth group received a standard drug cyclophosphamide (CP) 40 mg/kg p.o. or metranedazole (MTZ) 130 mg/kg p.o. or mercuric chloride (HgCl_2_) 0.864 mg/kg p.o. (as per the experiment requirment) whereas the fifth group kept as normal control and given only vehicle. Treatment was given up to five days and thereafter the results were obtained. The results on the bases of the data obtained revealed that BA is quite safe as it did not show any genotoxicity up to 500mg/kg dose. The three standard drugs used showed highly significant abnormal cytogenetic changes in comparison to the control group.

**450**

**Vitreous as a forensic specimen: Evaluation of vitreous levels of ethanol in acute overdosage in rabbits **

Arora B^1^, Velpandian T^1^, Ghose S^1^, Goyal VK^2^, Sharma M^2^

**^1^All India Institute of Medical Sciences, New Delhi, India; ^2^Forensic Science Laboratory, New Delhi, India.**

**Introduction:**

Ethanol is widely abused substance. Blood is subject to various postmortem changes so vitreous as a forensic specimen has been evaluated in this study.

**Methods:**

New Zealand albino rabbits weighing 1.5-2.5 kg and of either sex (n = 4) were orally given diluted ethanol at the dose of 10.49 ml/kg over the period of 25 minutes through an oral gavage. Amount of alcohol ingestion was noted incase if the rabbit died before 25 minutes while administering total dose. After death, the animal was left in a secured place at 25°C. Whole blood, plasma and vitreous samples were collected immediately after death and at 17hrs post death. Ocular tissues were also collected at 17 hrs. The samples collected were analyzed by head space Gas chromatography (GC).

**Results:**

Rabbits died at 3, 29, 72 and 80 minutes after the initiation of oral alcohol ingestion. Death at 3 and 29 min. marks absorption phase and 72 and 80 min. marks death in elimination phase. The gas chromatographic analysis revealed that 17 hours postmortem concentration of ethanol levels decreased to the extent of 20% (approximately) in all samples including vitreous. Along with the increasing duration between ethanol administration and death, the vitreous levels increased along with decrease in whole blood and plasma concentration.

**Conclusion:**

For death in absorptive phase, postmortem vitreous to whole blood ratio is 0.56±0.10 and for death in elimination phase ratio is 1.01±0.14. The study shows that vitreous may be a suitable forensic specimen along with blood. Further studies are in progress.

**451**

**Enhanced oxidative stress mediated toxicity in VL-17A cells due to alcohol and high glucose**

Karthikeyan C^1^, Chatterjee S^1^, Clemens DL^2^, Dey A^1^

**^1^AU-KBC Research Centre, Chennai, India; 2University of Nebraska Medical Center and Veterans Affairs Medical Center, Nebraska, United States.**

Oxidative stress plays a critical role in causing cellular dysfunction in pathophysiologic conditions like diabetes or alcoholism. Ethanol is metabolized in the liver by alcohol dehydrogenase (ADH) and ethanol inducible cytochrome P450 2E1 (CYP2E1) generates oxidative stress. As assessed by MTT cell viability assay, treatment of VL-17A cells with ethanol plus glucose resulted in greater toxicity when compared to HepG2 under the same conditions or VL-17A or HepG2 cells incubated in the absence or presence of ethanol or glucose alone. This decrease in viability, observed in ethanol plus glucose treated VL-17A cells was restored significantly by the addition of the antioxidants N-acetyl cysteine or trolox. Increased levels of reactive oxygen species (ROS) were also observed in VL-17A cells treated with ethanol plus glucose, which was greatly inhibited by the addition of N-acetyl cysteine or trolox. The oxidative stress observed in VL-17A cells treated with ethanol plus glucose was of much greater magnitude than untreated HepG2 or VL-17A cells or liver cells treated with either high glucose or ethanol, thus suggesting the role of oxidative stress in promoting alcohol plus high glucose induced toxicity in liver cells.

**452**

**Evaluation of duration dependent toxic effects of silica on lungs and lens**

Biswas S, Mondal KK, Lahiri SK

**R. G. Kar Medical College, Kolkata, India.**

Activation of inflammatory cells by silica releasing biochemical substances is a phenomenon in pulmonary silicosis. Toxic effects of silica on extrapulmonary tissues are also observed. A study was undertaken to evaluate silica induced duration dependent biochemical changes in bronchoalveolar lavage fluid (BALF) and toxic effects on lens. Pulmonary silicosis in rats was induced in acute model by single intratracheal injection of quartz dust, 10 mg in 0.05 ml saline, and in chronic model by inhalation of quartz dust with air, 40mg/m^3^ in simulation chamber. Control rats received vehicle only. According to duration of exposure each model was divided into 6 groups, 3 control vs 3 treated. Rats were sacrificed groupwise on 3^rd^, 5^th^ and 7^th^ day post exposure in acute cases and after 2, 4, and 8 weeks of SiO_2_ exposures in chronic cases. Protein, hydroxyproline, elastase and elastase inhibitory capacity (EIC) were estimated in BALF. Wet weight of lungs was measured. Malondialdehyde (MDA) was estimated in lens homogenates. Protein, hydroxyproline and elastase in BALF and lungs-weight were significantly (*P*< 0.001) raised in both models with gradually increasing levels. The EIC remained unchanged. Significant (*P*< 0.001) duration dependent increased MDA in lens homogenates was observed only in chronic model. Duration dependent biochemical changes in BALF were found responsible for initiation and progression of pulmonary silicosis. Chronic exposure to quartz may cause toxic injury to lens. Raised biochemical substances in BALF may serve as early markers for diagnosis of silicosis along with estimation of silica level in BALF

**453**

**Protective effect of alpha-ketoglutarate on heart against doxorubicin induced cardiotoxicity in rats**

Sharma M, Ashraf ZR, Pillai KK, Ahmad D, Imam F

**Jamia Hamdard (Hamdard University), New Delhi, India.**

The cytoprotective activity of alpha-ketoglutarate (alpha-KG) against doxorubicin (DOX) induced cardiotoxicity has been investigated. In present study, we evaluated the influence of doxorubicin on cellular defense mechanism against free radicals and the effect of alpha-KG supplementation on doxorubicin induced cardiotoxicity in Wistar rats. A single dose of doxorubicin (7.5 mg/kg i.v.) induced cardiotoxicity, manifested biochemically by significant elevation of serum creatine phosphokinase (CK) and lactate dehydrogenase (LDH). Cardiotoxicity was further confirmed by the significant increase in lipid peroxides, significant decrease in glutathione (GSH) levels, activity of catalase (CAT) and superoxide dismutase (SOD) in heart tissues. Alpha-KG (50 mg/kg p.o.) was administered 5 days before and 5 days after DOX injection. Protection was evaluated by measuring the changes in above biochemical parameters. Administration of alpha-KG (50 mg/ kg) offered a significant protection against cardiotoxicity induced by doxorubicin. The amelioration of cardiotoxicity was evident in significant reduction in serum CK and LDH. Alpha-KG (50 mg/ kg p.o) afforded cardioprotection by restoring the levels of above biochemical parameters within the normal range. Treatment with alpha-KG (50 mg/kg p.o.) in rats with DOX induced toxicity showed decrease in activity of serum enzymes CK and LDH; decrease in MDA levels along with restoration of GSH levels and activities of SOD and CAT. Cardioprotection was further confirmed by histopathogical studies, which demonstrated prevention of cytoplasmic vacuolar degeneration of cardiac myocytes. The present study reveals that oral administration of alpha-KG caused enhancement of endogenous myocardial antioxidants and prevents DOX induced myocardial oxidative stress.

**454**

**A study of toxicity of UNANI formulation in rats**

Nasiruddin M, Khan NA, Qasmi IA, Muzaffar M, Afzal M

**J. N. Medical College and A.K.Tibbiya Collge, A. M.U. Aligarh, India.**

**Introduction:**

To study toxic effect of Unani formulation used in Unani system of medicine.

**Materials and Methods:**

The study was conducted on male wistar rats weighing 100 – 150gms. They were divided into 3 groups of six animals each. They were provided standard diet and water ad libitum. Temperature 26±2°C and 12 hrs dark – light cycles were maintained. The animals in group 1^st^ were administered normal saline orally. The animals in groups 2^nd^ and 3^rd^ were administered alcoholic and aqueous extract of unani formulation Baladur in the dose of 10mg/100gm of animal per oral for 21 days respectively. On 22^nd^ day the animals were sacrificed and blood was collected for LFT and RFT estimation. The biochemical parameters were statistically compared and analyzed by student ‘t’ test.

**Results:**

The serum bilirubin, SGPT, SGOT, alkaline phosphatase and serum creatinine were 0.75 ± 0.01 mg/100ml, 17.30 ± 0.01 unit/ml, 21.25 ± 0.02 unit/ml, 7.74 ± 0.01 and 1.21 ± 0.01 respectively in group 1^st^. In group 2^nd^ serum bilirubin, SGPT, SGOT, alkaline phosphatase and serum creatinine were 0.69 ± 0.01 mg/100ml, 17.28 ± 0.01 unit/ml, 21.21 ± 0.02 unit/ml, 7.71 ± 0.01 and 1.21 ± 0.01 respectively. In group 3^rd^ serum bilirubin, SGPT, SGOT, alkaline phosphatase and serum creatinine were 0.70 ± 0.01 mg/100ml, 17.24 ± 0.01 unit/ml, 21.20 ± 0.02 unit/ml, 7.68 ± 0.01 and 1.19 ± 0.01 respectively. It was observed that the animals treated with unani formulation had statistically similar value as in control group (*P*>0.05).

**455**

**Compliance and detection of illicit drug use among opioid dependent subjects maintained on sublingual buprenorphine substitution therapy **

Jain R, Singh YPS

**All India Institute of Medical Sciences, New Delhi, India.**

**Introduction:**

A valid assessment of drug consumption is critical for evaluating substance abuse treatment programmes and interpreting treatment outcome data. Objective assessment has become essential for such programs. The study aims to evaluate the compliance and detection of illicit drug use among opioid dependent subjects maintained on sublingual buprenorphine substitution therapy.

**Methods:**

Male opioid dependent subjects being maintained on sublingual buprenorphine in a substitution program of an apex center were screened randomly for the presence of the illicit drugs use in their urine, using Thin Layer Chromatography, ELISA and the urine strip test. The compliance of buprenorphine was also corroborated using the urine analysis.

**Results:**

A total of 181 urine samples of patients maintained on sublingual buprenorphine were examined. The urinalysis showed 2.7% samples positive for opiates (morphine, codeine), 11.0% positive for dextroprpoxyphene, 2.2% positive for benzodiazepines (diazepam, nitrazepam) and 26% samples negative for all the medications.

**Conclusions:**

The data suggest that standard urine practices in buprenorphine substitution therapy may result in underestimates of the prevalence of illicit drug use. More frequent testing is desirable to ensure compliance and to screen the use of the illicit drugs in patients maintained on buprenorphine. (Supported by National Drug Dependence Treatment Centre, All India Institute of Medical Sciences, New Delhi, India).

**456**

**Teratological study of ‘Garbhpal Ras’, an ayurvedic formulation**

Mishra D^1^, Sinha M^1^, Kumar V^2^

**^1^Department of Prasuti Tantra, Institute of Medical Sciences, Banaras Hindu University, Varanasi, India; ^2^Department of Pharmaceutics, Institute of Technology, Banaras Hindu University, Varanasi, India.**

**Introduction:**

‘Garbhpal Ras’, an ayurvedic herbo-mineral formulation used during antenatal period for ensuring better nourishment to fetus, to prevent miscarriages and other minor ailments during pregnancy, subsequently results in birth of a healthy progeny. In present study ‘Garbhpal Ras’ was evaluated for teratogenic effects, if any, in pregnant rats (dams).

**Methods:**

Charles Foster albino rats with an average age about 120 days weighing 125±15 g were kept in isolation for 21 days to confirm that they are non-pregnant and had normal estrous cycle. Mating was accomplished by placing 3 female rats with 2 male rats in a cage overnight and mating was confirmed by the presence of sperms in the vaginal smear. Midnight of the day was considered as day zero of pregnancy. Twenty four dams were divided in four equal groups. ‘Garbhpal Ras’ was administered to dams per oral 3 and 10 times higher (12 and 40 mg/kg respectively) than the recommended dose from day six to day fifteen of pregnancy and compared with control (0.3% CMC). One of the groups administered with acetaldehyde (200 mg/kg). Acetaldehyde was used as standard foetotoxic and teratogenic agent. Rats used in study remain free from undesirable side effects or mortality.

**Results and Conclusion:**

Acetaldehyde administered dams showed fetotoxic effects like intrauterine growth retardation, shorter umbilical cord, intrauterine mortality and higher rate of fetal resorption while dams treated with vehicle or ‘Garbhpal Ras’ have not shown any such effects. Thus ‘Garbhpal Ras’ is safe for use during pregnancy in recommended therapeutic dose.

**457**

**Acute intoxication of cyclosporin caused by coadministration of decoction of *Pterocarpus marsupium* bark**

Makwana RK^1^, Harle UN^1^, Gaikwad NJ^2^

**^1^AISSMS College of Pharmacy, Pune, ^2^Department of Pharmaceutical Sciences, R.T.M. Nagpur University, Nagpur, India.**

In order to investigate the effect of *Pterocarpus marsupium* (PM) herb on cyclosporin absorption, disposition and toxicity rabbits were given cyclosporin (10 mg/kg) with or without decoction of bark. In a crossover design FPIA method was used to determine the blood concentration of cyclosporin. The decoction was characterized by their 1-epicatechin and marsupol contents. Our results indicated that the coadministration of PM significantly increased the C_max_ of cyclosporin by 67%. The AUC of cyclosporin was significantly elevated by 96% when coadministered with PM. Among the rabbits, 1/5 exhibits acute toxicity of cyclosporin after concomitant intake of PM, indicating interaction with commonly used drug.

**458**

**Possible role of alcoholic extract of roots of *Aerva javanica* in cisplatin and gentamicin - induced nephrotoxicity in rats**

Kumar V, Kaur P, Suttee A

**Lovely Professional University, Phagwara, India.**

**Introduction:**

The plant *Aerva javanica*, belonging to the family Amaranthaceae, is a perennial hairy - tomentose, erect to scandent, dioecious, conspicuous and undershrub. *Aerva javanica* root are reported to possess anthelmintic, diuretic, demulcent activity and is also used in the treatment of headache. The plant has also been documented earlier as therapeutic agent used in renal disorders.

**Methods:**

Hence the present study has been designed to evaluate the possible nephroprotective effect of the alcoholic extract of the roots of *Aerva javanica* in cisplatin and gentamicin-induced nephrotoxicity in rats. Animals were divided into seven groups i.e. group-I (control), group-II (cisplatin), group-III (gentamicin), group- IV (cisplatin + 200 mg/kg alcoholic extract of roots of *Aerva javanica* treated), group-V (gentamicin + 200 mg/kg alcoholic extract of roots of *Aerva javanica* treated), group-VI (cisplatin + 400 mg/kg alcoholic extract of roots of *Aerva javanica* treated), group-VII (gentamicin + 400 mg/kg alcoholic extract of roots of *Aerva javanica* treated).

**Results:**

The alcoholic extract of roots of *Aerva javanica* at both dose level i.e. 200 mg/kg and 400 mg/kg was found to normalize the changed blood urea, serum creatinine, total protein, serum albumin, urine volume, urine pH and bring about a marked recovery in kidney as evidenced microscopically.

**Conclusion:**

Hence it has been concluded that the roots of the plant *Aerva javanica* possesses marked nephroprotective activity with minimal toxicity and could have a promising role in the treatment of acute renal injury induced by nephrotoxin especially cisplatin and gentamicin.

**459**

**Tumorigenic property of filamentous cyanobacteria isolated from stagnant waterbody of Pilani**

Ashwin K, Ramani K, Verma SK

**Birla Institute of Technology and Science, Pilani, Rajasthan, India.**

**Introduction:**

Cyanobacteria (blue-green algae) are photosynthetic prokaryotes usually found in eutrophicated water. Some strains of cyanobacteria are responsible for bloom formation and in the production of bioactive compounds such as toxins, antibacterial, antifungal, antiviral, etc. The present study is aimed at biochemical analysis and tumourgenic activities of methanolic extracts of fresh water filamentatous heterocystous cyanobacteria isolated from the stagnant water body in the vicinity of Pilani, Jhunjhunu District of Rajasthan.

**Methods:**

Isolation and cultivation of cyanobacteria was carried out by broth and plate culturing techniques in BG11 media. The cultures were incubated at 27°C with illumination of 200 lux. The cells were harvested by centrifugation and were extracted with methanol. Toxins were partially purified by Thin-layer chromatography and subjected to protein estimation by Lowery’s methods. 100*µ*g/kg of extract was injected by i.p in swiss albino mice daily for 28 days. Toxicity of the extract was further confirmed by body weight analysis, biosopsy and TBARS assay of liver samples.

**Results:**

Tumors were observed in lower part of small intestine and colon in all animals. The results were further supported by bodyweight analysis and TBARS assay.

**Conclusion:**

The isolated strain of filamentous, heterocystous cyanobacteria found to have more amounts of toxic bioactive peptides that can cause colon tumors in mammalian models. The results of Lowey’s methods and TLC showed the presence of toxic peptides which are dependent on the nutritional factors such as nitrates in medium.

**460**

**Hepatoprotective activity of polyherbal formulation (Normeta^®^) on liver damage induced by alcohol, polyunsaturated fatty acids and iron in rats**

Gupta VP, Patere SN, Majumdar AS, Saraf MN

**Bombay College of Pharmacy, Mumbai, India.**

**Introduction:**

To evaluate the hepatoprotective action of Normeta^®^ on liver damage induced by alcohol, PUFA and iron in Wistar rats.

**Materials and Methods:**

Rats were divided into 8 groups (n=6). Group I: Vehicle control (VC), Group II: Alcohol 10-30% (Blood alcohol levels were maintained between 150-350mg/dl using head space gas chromatography), Group III: PUFA (15 % of diet) Group IV: Carbonyl iron (CI) (1.5-2% of diet), Group V (Stress control-SC): Alcohol, PUFA and CI, Group VI: Alcohol, PUFA and CI along with Silymarin (50mg/kg), Group VII: Alcohol, PUFA and CI along with Normeta^®^ (2ml/kg), Group VIII: Alcohol, PUFA and CI along with Normeta^®^ (4ml/kg). Serum parameter like serum glutamate pyruvate transaminase (SGPT), iron and proteins were determined. Liver parameters such as superoxide dismutase (SOD), catalase (CAT), Lipid peroxidation (LPO) were estimated. All the groups were treated p.o. for 30days.

**Results:**

Serum levels of iron, SGPT and liver LPO were significantly increased and levels of serum proteins, liver SOD and CAT activities were significantly decreased in SC group as compared to groups I, II, III and IV. In Normeta^®^ treated group serum iron, SGPT levels, liver LPO levels were decreased and serum proteins, liver SOD and CAT activities were increased as compared to SC group.

**Conclusion:**

The study revealed significant exacerbation of the alcohol induced oxidative stress in the presence of PUFA and iron. The effects of Normeta^®^ on physico-metabolic parameters were comparable with silymarin and indicated that Normeta^®^ has favourable effect in bringing down the severity of hepatotoxicity.

**461**

**Study of drug poisoning cases reported to national poisons information centre, AIIMS, New Delhi**

Peshin Sharda S, Gupta Yogendra Kumar

**National Poisons Information Centre, Department of Pharmacology, All India Institute of Medical Sciences, New Delhi, India.**

The aim of the retrospective study was to determine the incidence of poisoning due to various categories of drugs as reported to the National Poisons Information Centre (NPIC), All India Institute of Medical Sciences, New Delhi, India. The enquiries about various aspects of poisoning were made on telephone by professionals and information recorded on a preset proforma. Required information was conveyed by a follow-up call after consulting relevant literature. Analysis of eight years data (1999-2007), revealed that a number of products were implicated in poisoning, namely household products (42.57%), drugs (19.25%), agricultural pesticides (13.11%). Industrial chemicals (10.81%), bites and stings (4.57%), plants (2.16%), unknown and miscellaneous products (3.33%, 4.29%) respectively. The incidence of poisoning due to drugs was (19.25%) with 99.19% calls regarding the management of cases and 0.8% seeking information on various aspects of drugs. Preponderance of males over females was noted (M=57.95%, F=42.04%).Accidental poisoning was the common mode (55.39%) followed by suicidal (43.86%) and unknown mode (0.75%) and the route of exposure was exclusively oral. Various groups of commonly ingested drugs included benzodiazepines(18.66%), anticonvulsants (11.92%), analgesics (10.17%), antihistamines (7.47%) besides others. Ingestion of combination of various drugs was also seen (5.05%). A glaring feature of the study was ingestion of drugs by children contributing 45.12% to the total number of cases involving drug ingestion. Children below the age of 6 years were mainly involved (71.55%).Drugs commonly implicated in children included anticonvulsants (12.14%), benzodiazepines (10.07%), analgesics (9.62%), antihistamines(6.37%), besides others. Implementation of proper preventive measures are the key challenges for achieving poisons control that can reduce morbidity and mortality.

**462**

**Toxicokinetic and recovery of ACTP (triclopyrbulotyl) ester in goats folliwng single oral dose administration**

Bagchi, B^1^, Sar TK^1^, Mandal, TK^1^, Chakraborty, A^1^, Bhattacharyya A^2^, Chowdhury A^2^

**^1^Department of Pharmacology and Toxicology, 37 and 68 K.B. Sarani, Kolkta, ^2^Department of Agricultural Chemicals, Bidhan Chandra Krishi Viswavidyalaya, Mohanpur, Nadia, India.**

Toxicokinetics including metabolism and recovery of ACTP ester, a selective systemic herbicide was studied in 24 healthy adult male and female black Bengal goats after oral administration of ACTP ester by stomach tube after suspending in 1% carboxymethyl cellulose (CMS) at 400 mg/kg. The dose level was fixed at 400 mg/kg by trial and error as the treated animals showed some toxic signs which progressively increased till 6hr pd and all the toxic signs waned out by 24 hr pd. Estimation of ACTP ester and its two metabolites namely triclopyr acid and trichloropyridinol from different substrates was done by HPLC. The maximum concentration of ACTP ester appeared in blood at 6 hr, declined gradually and persisted upt 60 hr and the kinetic behaviour followed a ‘two compartment open model’. Maximum amount of ACTP ester excreted at 24-48 hr through faeces and urine. But excretion of ACTP ester through urine was completed by 96 hr, while it was excreted through faeces till 168 hr pd. Two major metabolites of ACTP ester namely triclopyr acid and trichloropyridinol were identified from faeces, urine, gastrointestinal contents and tissues of goats. Maximum quantity of triclopyr acid and trichloropyridinol were excreted through faeces at 24 and 48 hr pd and excretion was continued till 168 hr. Both the metabolites started to excrete through urine by 24 hr and continued to be excreted till 168 hr pd. Maximum ACTP ester residue was recovered from tissues of goats slaughtered after 4 days followed by 5, 6 and 7 days in sequence. The total recovery perentage of ACTP ester and its metabolites in terms of parent compound recovered from faeces, urine, gastrointestinal contents and tissues of goats were respectively 4.49, 42.26, 1.72 and 16.10 after 4 day pd. 4.72, 47.32, 1.27 and 13.55 after 5 day pd, 5.35, 50.65, 1.33 and 12.00 after 6 day pd and 5.34, 51.73, 1.27 ad 10.30 after 7 day pd.

**463**

**Long term toxicity study of endosulfan in black bengal goats after repeated oral administration**

P Subramanian, Datta Bakul Kr., Chakraborty Aditi, Karmakar Utpal, Sar TK, Chakraborty Animesh Kr., Tapan Kr. Mandal, Bhartachariya Anjan 

**West Bengal University of Animal and Fishery Sciences, Nadia, West Bengal, India.**

**Objective:**

The present study was undertaken to find out long term effect of endosulfan in black Bengal goats after repeated oral administration.

**Materials and Methods:**

Twelve black Bengal adult goats of either sex were divided into two groups each containing three male and three female. The first group was kept as control and second group was considered as experimental which was received endosulfan orally at the rate of 2.6 mg kg^-1^ body weight for 56 days. Blood samples were collected at every 0, 7, 14, 28, 35, 42, 49 and 56 days of administration. From blood samples, glucose, protein, AST, ALT, Serum IgG and residue of endosulfan α, β and sulfate were estimated. On day 57, three animals from each group were sacrificed. The remaining were sacrificed on 14 days after last dosing of endosulfan. Various organs were collected from control and experimental groups and utilized for histopathology and estimation of residual status of endosulfan α, β and sulfate isomer. Part of liver tissue of both groups was also utilized for estimation of antioxidant status and cytochrome P_450_.

**Results and Discussion:**

Increased blood glucose level and decreased reduced glutathione, and catalase activity were observed in experimental groups where as serum protein, AST, ALT, IgG level and lipid peroxidation and SOD content were not altered significantly. Cytochrome P_450_ content of liver microsomal pellet of experimental goats were increased significantly compared to control goats. Residual status showed most of the organ contained endosulfon α, β and sulfate concentration. Only β and sulfate isomers were not detected in bone, brain, skin and lymphnode. Tissue residue of endosulfan α were detected in liver, kidney and ovary in goats sacrificed after 14 days of recovery period. Similarly, β and sulfate isomers were only detected in lungs and kidney respectivey. Histopathological study showed mild pathological alteration in lung, liver, kidney, testis and ovary tissues.

**Conclusion:**

Continuous oral administration of endosulfan at 2.6 mg kg^-1^ b.wt. Showed mild toxicity in goats, and its withdrawal period should not be less than 14 days.

**464**

**Neuroprotective role of coenzyme Q10 in polychlorinated biphenyl induced oxidative stress in mice brain**

Kamble R Y, Jadhav S R, and Majumdar A S

**Department of Pharmacology, Bombay College of Pharmacy, Mumbai, India.**

**Objective:**

To determine the neuroprotective role of Coenzyme Q10 (CoQ10) against Polychlorinated Biphenyl (PCB) induced oxidative stress in brain of Swiss albino mice.

Materials and Methods:

Mice were divided into 4 groups (n=6). Group I: vehicle control, Group II: PCB (5 mg/kg/day, p.o). Group III: PCB (5 mg/kg/day, p.o) along with Coenzyme Q10 (10 mg/kg p.o) Group IV: PCB (5 mg/kg/day, p.o) along L- deprenyl (1 mg/kg i.p.). All groups were treated once a day for 28 days. At the end mice were sacrificed and the brain was removed. Parameters such as superoxide dismutase (SOD), catalase (CAT), glutathione peroxidase (GPx), acetylcholinesterase (Ache) and reduced glutathione (GSH) were estimated. Lipid peroxidation (LPO) was determined.

Results:

The % decrease in SOD, CAT, GPx, AChe and GSH was 47.4%, 45.6%, 19.8%, 59.3 % and 67% respectively in PCB treated group. CoQ10 administration along with PCB retrieved all the parameters significantly. In CoQ10 treated group SOD, CAT, GPx, AChe and GSH was restored upto 63.17%, 88.93%, 35.15%, 87.4% and 87.67% respectively. PCB raised LPO by 100% as compared to control which in presence of CoQ10 was restricted to 11%. Results were analysed using Student’s t-test.

Conclusion:

Polychlorinated Biphenyls, known persistent organic pollutant (POP) induce oxidative stress in mice brain by decreasing the activities of antioxidant enzymes, which can be protected by Coenzyme Q10 treatment. This study shows usefulness of Coenzyme Q10 as supplemental nutritional therapeutic agent in disorders affecting the brain free radical metabolism.

**465**

**Antioxidant actions of asparagus racemosus against iron-induced oxidative stress in broilers**

Ramakrishnan V, Gopala Reddy A, Kalakumar B, Somasekhar Reddy K

**College of veterinary science,Hyderabad, India**

Experimental study was carried out to evaluate the protective anti-oxidant potential of Asparagus racemosus against ironinduced oxidative stress in poultry. A total of 45, day old male broiler chicks were randomly divided into three groups with fifteen birds in each group. Group 1 was maintained on basal diet and group 2 on FeSO4 at 0.5% of feed 6 weeks (42 days). Group 3 was given FeSO4 containing diet for the first 4 weeks (28 days) and subsequentaly treated with Asparagus racemosus at 0.1% level in feed till the end of the 6th week. The performance parameters were recorded at weekly intervals. Anti-oxidant defense profile, biomarkers of renal damage and biomarkers of hapatic damage were estimated at the end of 4th and 6th week. Histopathological studies and estimation of TBARS and GSH were done at the end of 6th week. Iron treatment resulted in significant (P<0.05) reduction in body weights and GSH (6th week), while TBARS (6th week), SOD, catalase, ALT and creatinine were significantly (p<0.05) increased at the end of 4th week in groups 2 and 3, and all these parameters exhibited similar trend at the end of 6th week in iron toxic control group 2. Following treatment, there was a marked improvement in all the above parameters in-group 3 as compared to the toxic control group 2 at the end of 6th week. Histological abnormalities were also recorded in the toxic control group 2, while the treated group revealed lesions of mild intensity or signs of regeneration. Thus, it is concluded that iron induces biological damage by means of oxidative stress and Asparagus racemosus offered better amelioration.

**466**

**Toxicokinetics and recovery studies of dicamba dimethyl amine salt (herbicide) in goats following single oral administration**

Mukherjee Madhusudan, M Prashant, Datta Bakul Kr., Sar TK, Chakraborty AK, TK Mandal

**West Bengal university animal and fishery sciences, Kolkota, India.**

**Objective:**

Dicamba dimethyl amine salt a herbicide used in field to control weeds. Due to accidental ingestion through farm animal it may affect human health through food chain. The present study was undertaken to identify toxicokinetics and residual properly of Dicamba dimthyl amine salt in black Bengal goats.

**Materials and Methods:**

Ten black Bengal goats of either sex were divided into two groups. Group I consisted of one male and female goats served as control and group II consisted of 4 male and 4 female goats which were received Dicamba dimethyl amine salt as single oral administration at the rate of 1400mg kg-1. Blood samples were collected at different time interval to study toxicokinetic parameters. Urine and feces samples were also collected at different time interval. After 4^th^ day four animals of group II were sacrificed and remaining four animal were sacrificed on day 7^th^ to study total recovery of Dicamba Dimethyl amine salt from tissue by gas chromatography.

**Result and Discussion:**

An adequate level of Dicamba Dimethyl amine salt (DDAS) was defected in blood at 5 min followed by gradual increase in concentration and maximum concentration achieved at 15 min and minimum concentration was detected at 36 hr. Short absorption half life suggested its quick absorption and slow elimination properly. Higher Vd_area_ values also indicated wide distribution properly of DDAS in body. From urine and feces significant amount of DDAS were excreted. Residual concentration of DDAS could be detected in all the tissue of goats slaughtered on day 4^th^ whilst DDAS could not be detected in most tissues slaughtered on day 7^th^.

**Conclusion:**

Dicamba Dimethyl amine salt has lower affinity to accumulate in tissues after single dose oral administration and goat meat should take after 7 days of DDAS exposure.

**467**

**Toxicological screening of an indigenous polyherbal formulation (SPF07) in experimental animals**

Gite MS, Desai SK, Gawali VS, Naik AB, D.souza LL

**Principal K.M.Kundnani College of Pharmacy., Mumbai, India.**

**Introduction:**

Acute and sub-acute toxicity study of an indigenous polyherbal formulation (SPF07) employed for is antianxiety activity was carried out in albino Wistar rats to assess its safety by evaluating effects morphological, gross behavioral, body weight changes as well as determining the histopathological, biochemical and hematological changes.

**Methods:**

Albino Wistar rats of both sexes in the weight range of 150-200g were procured from Bharat Serum and Vaccines, Thane and maintained on standard pellet and water diet. All reagents and chemicals used were of analytical grade. All procedures carried out were under the approval of Institutional Animal Ethics Committee. The test formulation (250, 500 and 750mg/kg) was administered p.o. once daily for 28 days. Cage side observations, changes in morphology, behaviour and body weight were noted weekly. At the end of the exposure period the animals were humanely sacrificed and biochemical, hematological and pathological changes (macroscopic and microscopic) were determined.

**Results:**

Based on biochemical analysis of renal and hepato-biliary functions, such as the level of urea, creatinine, transaminases and alkaline phosphatase, we were able to conclude that the indigenous polyherbal formulation SPF07 is generally well tolerated by rats. This was also confirmed by hematological and histopathological examination. SPF07 was found to be safe in acute and subacute toxicity studies where as chronic toxicity studies are further required for the support of the safe and sound use of this polyherbal formulation for recommended prolonged use.

**Conclusion:**

The polyherbal drug (SPF07) is devoid of toxic effects in rats upto the dose of 750 mg/kg which is 4 times higher than the recommended dose.

**468**

***In vivo* genotoxicity studies of gatifloxacin**

Patel NG^1^, Tripathi P^2^, Patel NJ^2^, Panchal AH^2^, Vyas AS^2^

**^1^Sigma Institute of Pharmacy, Vadodara, India; ^2^S.K.Patel College of Pharmaceutical Education and Research, Mehsana, India.**

**Objective:**

The *in vivo* genotoxicity of gatifloxacin, a diflourinated antibacterial drug, was evaluated by employing mouse *in vivo* chromosomal aberration test in bone marrow cells.

**Materials and Methods:**

Mice (6–8 weeks old) were segregated into 10 groups (n=6). Two groups of animals were injected distilled water intraperitoneally for use as vehicle control. Another two groups were administered cyclophosphamide (10 mg/kg) and the remaining six groups of animals were administered gatifloxacin (33.3, 66.6 and 133.3 mg/kg), two groups for each dose through the same route. Animals were sacrificed 12 and 24 h after treatment by cervical dislocation. Ninety minutes before sacrifice animals were injected 0.2 ml of colchicine (4 mg/kg). Bone marrow was flushed in 0.056 % KCl, centrifuged for 10 min and fixed in cold fixative. Centrifugation and fixation were repeated three times at an interval of 15 min. Finally, the cells were resuspended on clean chilled slides, flame dried and stained in 10% buffered giemsa stain. Data were evaluated as percent aberrant metaphase cells (percentage of abnormal metaphase excluding gaps) and chromosomal aberrations per cell (excluding gaps). Results were analyzed by unpaired twotailed student’s t-test.

**Result:**

Statistically significant reduction in mitotic index, increase in chromosomal aberrations and percent abnormal metaphase was observed at the highest dose (133.3 mg\ kg) of the drug. This increase in percentage of damage was lesser than the positive control group while more than the control group.

**Conclusion:**

These results seem to indicate that gatifloxacin is a weak clastogen in the bone marrow cells.

**469**

**Role of β-carotene and N-acetyl-L-cysteine with and without praziquantel treatment in modulating *Schistosoma mansoni*-induced genotoxic effects on albino mice**

Ebeid F^1^, El-Lakkany N^1^, Seif el-Din S^1^, Hagag H^2^

**Theodor Bilharz Research Institute^1^, Imbaba,; Faculty of medicine^2^, Cairo University, Egypt.**

**Introduction:**

This study investigates the potential genotoxicity of *Schistosoma mansoni* infection in mice and the possible protective role of the antioxidants pro vitamin beta-carotene (BC) and N-acetyl- L-cysteine (NAC) with and without praziquantel (PZQ) treatment against these genotoxic effects.

**Materials and Methods:**

Mice were classified into eight groups; the 1^st^ and the 2^nd^ groups served as normal and infected untreated controls. Groups III and IV were treated 7 weeks post infection with PZQ in a full (500X2 mg/kg) or PZQED_50_ (74.64 mg/kg) doses. The remaining 4 groups were infected and treated with BC (2.7 mg/kg) or NAC (300 mg/kg) for one week before infection and the last two groups were treated by BC or NAC in addition to PZQ ED_50_. Animals were sacrificed two weeks following the last dose of PZQ. Animals were injected each intraperitonealy with 5 mg/kg colchicine 2 hours before sacrifice. Bone marrow from both femurs of each mouse was separated. For each mouse 50 metaphase spreads were examined microscopically for chromosomal aberrations and another 25 second division cells were examined for sister chromatid exchanges (SCEs). Cell cycle kinetics and mitotic indices were also determined.

**Results:**

Revealed that schistosomiasis is genotoxic as shown by the significant increase in both the percentage of structural chromosomal aberrations and the frequency of SCEs. Moreover, it inhibited cell division and caused cell cycle delay expressed by replicative index and average generation time (AGT). Administration of either BC or NAC alone decreased significantly the number of breaks, the total structural chromosomal aberrations, and the frequency of SCEs as compared with the infected untreated group. In addition, BC or NAC produced a significant decrease in the AGT and mitotic index. Addition of PZQ to BC or NAC did not enhance the antimutagenic effects of these antioxidants significantly. In conclusion, schistosomiasis induces genotoxic effects in mice and the use of the antioxidants BC or NAC could be considered a promising approach toward inhibiting the schistosome-induced cytogenetic damage.

**470**

**Sub acute toxicity study of antiretroviral drug-azidothymidine in rats**

Kumari B^1^, Singh RK^2^, Bansode FW^2^

**^1^University of Rajasthan, Jaipur, India; ^2^Central Drug Research Institute, Lucknow, India.**

Now days, AIDS is transformed into a universal scientific tenet. AIDS is caused by a Retrovirus called Human Immunodeficiency Virus (HIV). AZT (Azidothymidine- commercial name Zidovudine) is the first drug licensed as anti-retroviral. It is a nucleoside (thymidine) analogue that interferes with transcription of single stranded viral RNA into double stranded DNA by inhibiting the reverse transcriptase enzyme but this drug has some side effects. In our laboratory 10 rats of Charles Foster strain taken from CDRI breeding colony were divided into two groups- IandII. Group II was orally administered the drug AZT at the dose level of 60 mg/kg body weight daily and observed for 28 days. Group I was given distilled water as equal volume of vehicle used with the drug given to group II rats. After completion of the duration, The effect on body weight, general behavior, food and water intakes, haematological parameters –TLC, DLC, T-RBC, *µ*RBC, MRBC, Hb, MHC, MCHC, T-PLT, MPLT, MPV, and Hct were monitored and after that autopsy of the above rats was done. Different organs like liver, kidney, spleen, heart, testis and ovary were taken out and kept in 10% formal saline solution, and then they were processed like- block making, section cutting, and staining with H andE for histopathological observations. In drug treated rats, histopathological changes were seen.

